# Mechanisms of Action of *trans* Fatty Acids

**DOI:** 10.1093/advances/nmz125

**Published:** 2019-11-29

**Authors:** Antwi-Boasiako Oteng, Sander Kersten

**Affiliations:** Nutrition, Metabolism and Genomics Group, Division of Human Nutrition and Health, Wageningen University, Wageningen, The Netherlands

**Keywords:** elaidic acid, industrial *trans* fatty acid, ruminant *trans* fatty acid, inflammation, ER stress, lipid metabolism, cholesterogenesis, cardiometabolic disease

## Abstract

Human studies have established a positive association between the intake of industrial *trans* fatty acids and the development of cardiovascular diseases, leading several countries to enact laws that restrict the presence of industrial *trans* fatty acids in food products. However, *trans* fatty acids cannot be completely eliminated from the human diet since they are also naturally present in meat and dairy products of ruminant animals. Moreover, bans on industrial *trans* fatty acids have not yet been instituted in all countries. The epidemiological evidence against *trans* fatty acids by far overshadows mechanistic insights that may explain how *trans* fatty acids achieve their damaging effects. This review focuses on the mechanisms that underlie the deleterious effects of *trans* fatty acids by juxtaposing effects of *trans* fatty acids against those of *cis*-unsaturated fatty acids and saturated fatty acids (SFAs). This review also carefully explores the argument that ruminant *trans* fatty acids have differential effects from industrial *trans* fatty acids. Overall, in vivo and in vitro studies demonstrate that industrial *trans* fatty acids promote inflammation and endoplasmic reticulum (ER) stress, although to a lesser degree than SFAs, whereas *cis*-unsaturated fatty acids are protective against ER stress and inflammation. Additionally, industrial *trans* fatty acids promote fat storage in the liver at the expense of adipose tissue compared with *cis*-unsaturated fatty acids and SFAs. In cultured hepatocytes and adipocytes, industrial *trans* fatty acids, but not *cis*-unsaturated fatty acids or SFAs, stimulate the cholesterol synthesis pathway by activating sterol regulatory element binding protein (SREBP) 2–mediated gene regulation. Interestingly, although industrial and ruminant *trans* fatty acids show similar effects on human plasma lipoproteins, in preclinical models, only industrial *trans* fatty acids promote inflammation, ER stress, and cholesterol synthesis. Overall, clearer insight into the molecular mechanisms of action of *trans* fatty acids may create new therapeutic windows for the treatment of diseases characterized by disrupted lipid metabolism.

## Introduction

*trans* Fatty acids are unsaturated fatty acids that contain 1 or more unconjugated double bond in the *trans* configuration. The term *trans* fats is used to describe triglycerides that are rich in *trans* fatty acids. Although some *trans* fatty acids are produced during fermentation in the rumen of ruminant animals, most *trans* fatty acids are generated during industrial processing through partial hydrogenation of vegetable oils rich in PUFAs. The amount of *trans* fatty acids in partially hydrogenated vegetable oils can be as high as 60%, with different isoforms of *trans*-octadecenoic acid (*trans* 18:1) accounting for 80–90% of the total *trans* fatty acid content (1–3). Foods containing these industrially produced artificial *trans* fatty acids carry several benefits including improved texture, better taste, and enhanced shelf life (4–6).

**FIGURE 1:**
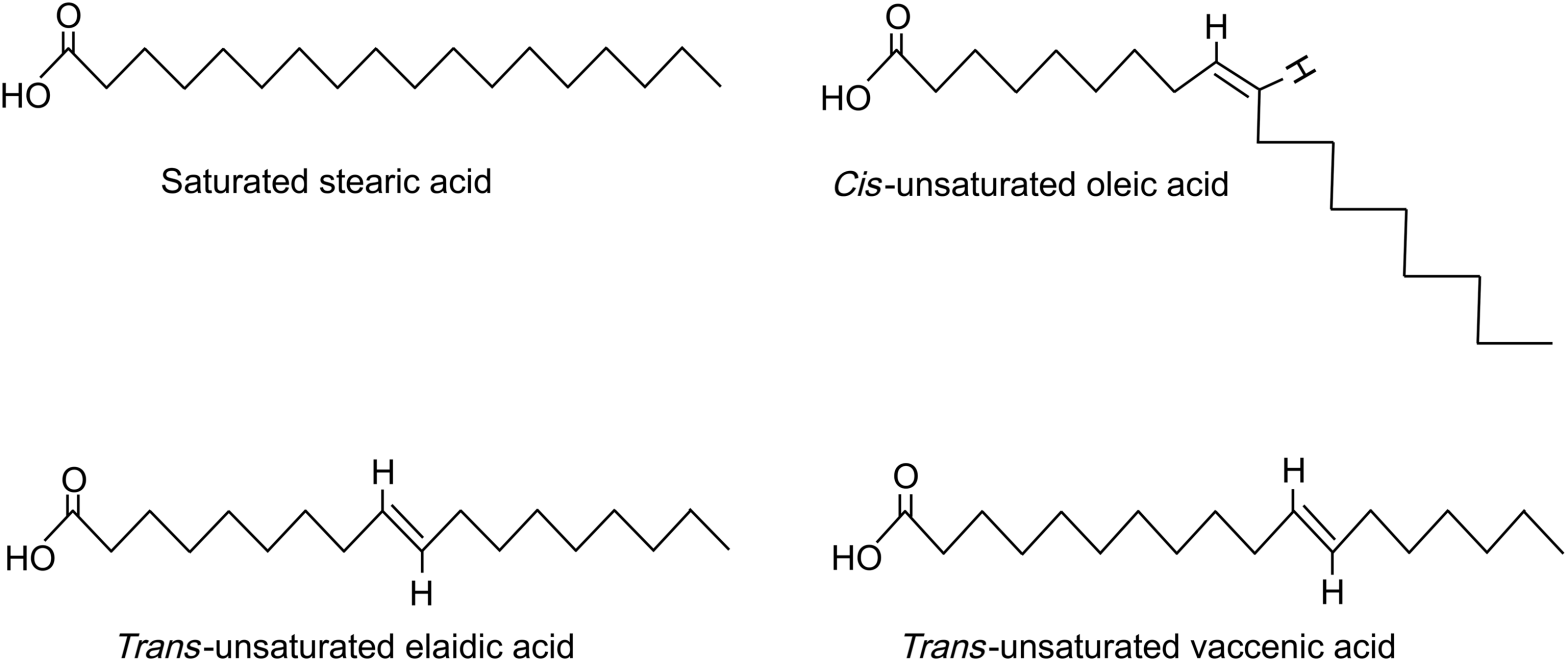
Structure of the geometric isomers of C18 fatty acids showing elaidic and vaccenic acids with a *trans* double bond, oleic acid with a *cis* double bond, and the fully saturated stearic acid.

As indicated above, *trans* fatty acids are defined by the presence of 1 or more unconjugated *trans* double bond. Fatty acids that contain conjugated *trans* double bonds, such as conjugated linoleic acid, are considered a separate entity and are only covered briefly in this review. In the *trans* configuration, the 2 hydrogen atoms around the double bond point in opposite directions, whereas in the *cis* configuration these hydrogen atoms point in the same direction. In comparison to the *cis* form, in which the 2 bond angles add up to create a kink in the alkyl chain, in the *trans* form, the 2 bond angles correct each other, giving rise to a straight chain tertiary structure akin to that of SFAs (**Figure 1**) (5, 7). Differences in tertiary structure affect crystalline packaging, which in turn influences physicochemical properties such as the melting point. For example, the 18-carbon *cis* oleic acid is liquid at room temperature, with a melting point of 14°C. By contrast, elaidic acid, which is the *trans* geometric isomer of oleic acid, has a much higher melting point at 45°C and is solid at room temperature. For comparison, the fully saturated stearic acid has a melting point of 69°C (8). As discussed below, evidence abounds indicating that beyond affecting their geometric isomerization and physicochemical properties, the *cis*-*trans* configuration of fatty acids has a major influence on the physiological properties after human consumption.

Evidence on the potential detrimental effects of industrial *trans* fatty acids first emerged in the 1950s. In 1957 Kummerow and colleagues (9) found that lipid extracts of tissue specimens from 24 human subjects who died of heart disease contained ≤12.2% *trans* fatty acids in their adipose tissue, 14.4% in the liver, 9.3% in heart tissue, 8.8% in aortic tissue, and 8.8% in atheroma. Subsequent studies showed that the concentration of *trans* 18:1 and 16:1 fatty acids was 6.8% higher in the adipose tissue of individuals who died of ischemic heart disease compared with individuals who died of other causes (10, 11). In 1990 Mensink and Katan (12) firmly demonstrated the plasma cholesterol-raising effect of industrially produced *trans*-octadecenoic acids in human volunteers. Several years later Willett and colleagues (13) found in the Nurses’ Health Study that the intake of *trans* fatty acids from partially hydrogenated vegetable oils, after adjustment for age and total energy intake, was positively associated with the risk of coronary artery disease, with the RR of the highest versus lowest quintile at 1.50 (95% CI: 1.12–2.00, *P* = 0.001).

The studies that strongly connected the intake of *trans* fatty acids with cardiovascular derailment were initially abhorred by players in the margarine industry. However, this later fueled more research into the potential atherogenicity of *trans* fatty acids. Collectively, these studies strongly suggest a causal relation between industrial *trans* fatty acid consumption and the development of cardiovascular disease in humans (13–18). In response to this finding, a number of countries enacted laws that either restricted or completely banned food companies from incorporating *trans* fatty acids into their food products. Two independent studies found that New York State counties that had 3 or more years of restrictions of industrial *trans* fatty acids had an average 7.8% reduction in the incidence of myocardial infarction and a 4.5% reduction in cardiovascular disease mortality rates compared with counties with no such restrictions (19, 20).

In many countries food items such as margarine, crackers, bakery products, cookies, and deep-fried foods were previously loaded with industrial *trans* fatty acids. Nowadays however, the concentrations of *trans* fatty acids in these foods are very low, which has resulted in a substantial decline in the intake of industrial *trans* fatty acids (21–30). For instance, the 2018 Dutch Nutrition Survey reported that in 2018, *trans* fatty acids only provided ∼0.3% of the daily energy requirement, as opposed to 5–10% several decades ago. However, industrial *trans* fatty acids persist in our food supply because laws aimed at restricting industrial *trans* fatty acids have not yet been instituted in every country. In addition, *trans* fatty acids cannot be completely removed from human diets due to their presence in the meat and dairy products of ruminants. These *trans* fatty acids, which include *trans*-vaccenic acid and rumenic acid, are generated through the biohydrogenation of PUFAs in the rumen of these animals (1, 31, 32). Ruminant fats can contain ≤8% of *trans* fatty acids. *Trans*-vaccenic acid is the predominant form and represents 50–80% of the total ruminant *trans* fatty acid intake (29, 32, 33).

As health concerns connected to the consumption of industrial *trans* fatty acids can be mitigated by removing them from foods, there has been little incentive for researchers to probe mechanistic insights related to *trans* fatty acids. The end result is a marked imbalance between the vast amount of epidemiological data on *trans* fatty acids and health outcomes and the limited understanding of the molecular mode of action of *trans* fatty acids. By collating the mechanistic studies on *trans* fatty acids, this review aims to improve our understanding of the mechanisms underlying the deleterious effects of *trans* fatty acids. The focus is on the physiological, metabolic, and molecular pathways affected by *trans* fatty acids. To achieve this objective, some parallels will be drawn between *trans* fatty acids and other relevant types of fatty acids. In addition, this review will carefully dissect the argument that ruminant *trans* fatty acids have contrasting effects to industrial *trans* fatty acids.

## Potential Adverse Outcome Pathways of *trans* Fatty Acids

Although the majority of studies performed on *trans* fatty acids are observational, a substantial number of experimental studies have been performed. These studies vary from experiments in cultured cells and animal models to human clinical trials. A number of these studies have provided evidence that certain *trans* fatty acids influence the regulation of physiological processes such as lipid metabolism, inflammation, oxidative stress, endoplasmic reticulum (ER) stress, autophagy, and apoptosis. The dysregulation of some of these biological pathways by *trans* fatty acids has been proposed as a potential underlying mechanism that contributes to the negative effects of *trans* fatty acids on cardiometabolic health (34–36). These potential adverse outcome pathways are further explained in detail in the following sections.

## Plasma Cholesterol and Lipoprotein Profile

Cholesterol is transported in the blood mainly as part of LDL and HDL. Smaller amounts of cholesterol are also contained in the triglyceride-rich chylomicrons, VLDL, and their remnant particles. Increased concentrations of LDL cholesterol, non-HDL cholesterol, or apoB-containing particles increase the risk of atherosclerotic cardiovascular disease (37–39).

Several studies have examined the effect of *trans* fatty acids on plasma cholesterol concentrations and lipoprotein dynamics. A randomized crossover trial carried out at Wageningen University in 1990 randomly assigned 59 participants to each of 3 isocaloric diets for 3 wk in which 10% of the daily energy was provided by either oleic acid, *trans* isomers of octadecenoic acid, or palmitic and lauric acid. When compared with the oleic acid diet, the *trans* fatty acid diet significantly reduced the serum concentrations of HDL cholesterol by 12%, and increased the concentrations of total and LDL cholesterol by 5.8% and 13.9%, respectively. By contrast, the SFA diet did not significantly affect serum HDL cholesterol but significantly raised the concentrations of total and LDL cholesterol by 12.1% and 17.6%, respectively (12). Significant changes in plasma lipoproteins were also recorded in a similar crossover study conducted in 56 participants. Mean serum concentrations of LDL cholesterol were 6.0% higher after an SFA-enriched diet than after a *cis*-unsaturated fatty acid diet, and 8.4% higher after a *trans*-unsaturated fatty acid diet. Total serum cholesterol concentrations were 3.2% and 3.4% higher after the saturated and *trans*-unsaturated diets, respectively, compared with the *cis*-unsaturated diet. Meanwhile, HDL cholesterol concentrations were 4.1% and 6.8% lower after the saturated and *trans*-unsaturated diets, respectively, than after the *cis*-unsaturated diet (40). This unique property of industrial *trans* fatty acids to simultaneously increase the circulating concentrations of total and LDL cholesterol whilst decreasing the concentrations of HDL cholesterol is believed to result in stronger atherogenicity compared with saturated or *cis*-unsaturated fatty acids.

Several additional studies examined the ability of industrial *trans* fatty acids to modulate plasma cholesterol. In a randomized crossover study, 32 healthy men and women were assigned for 4 wk to either a diet providing 9.2% energy as industrial *trans* fatty acids from partially hydrogenated soybean oil or 12.9% energy as SFAs from palm kernel fat. The *trans* fatty acid diet decreased serum HDL cholesterol concentrations by 19% yet did not significantly affect LDL cholesterol and triglyceride concentrations in comparison with the SFA diet (41). In another crossover study with each dietary intervention lasting 5 wk, 50 normocholesterolemic men consumed 8% of daily energy as either *trans* 18:1 or stearic acid. Compared with the stearic acid diet, the *trans* fatty acid diet resulted in significantly higher plasma concentrations of total (0.22 mM, 4.5%) and LDL cholesterol (0.26 mM, 8.35%), with no significant differences in HDL cholesterol concentrations (42).

Mauger and colleagues (43) reported that the athero-genicity of *trans* fatty acids may lie in part in their ability to reduce LDL particle size in a dose-dependent manner. Matthan and colleagues (44) have likewise shown in hypercholesterolemic women that the deleterious lipoprotein profile caused by *trans* fatty acid intake is partly explained by increased apoA1 and decreased apoB100 catabolism. Interestingly, a limited number of studies did not find a significant deleterious effect of *trans* fatty acid intake on plasma lipid concentrations (45, 46). These discrepant results are potentially due to the use of a different reference or control diet. Additionally, differences in amounts of *trans* fatty acids consumed may account for some of the conflicting findings, as the effects of *trans* fatty acids on plasma lipids seem proportional to intake (47).

Studies performed in rodent models support the plasma LDL-raising effect of *trans* fatty acids. In LDL-receptor knockout mice, provision for 16 wk of a diet enriched with 34.7 g of elaidic acid from partially hydrogenated soybean oil per 100 g/fat increased plasma concentrations of total cholesterol, LDL cholesterol, and triglycerides by 5.5-fold, 3.2-fold, and 3.8-fold, respectively, when compared with an isocaloric diet rich in PUFAs. Concentrations of plasma HDL cholesterol did not differ significantly (48). A similar but independent study performed under the same experimental conditions in the same animal model also resulted in significant increases in plasma concentrations of total cholesterol by 2.1-fold, LDL cholesterol by 1.7-fold, triglycerides by 4.2-fold, and a reduction in HDL cholesterol by 2.3-fold (49). Overall, it can be concluded that the positive association between *trans* fatty acid intake and cardiovascular disease risk is likely, at least in part, explained by the unfavorable effect of *trans* fatty acids on plasma LDL cholesterol and the overall lipoprotein profile.

## Inflammation

Sterile inflammation describes inflammation that occurs in the absence of an infection and is a symptom of numerous chronic diseases and pathologies, including atherosclerosis. Indeed, in addition to being a lipid-driven process, atherosclerosis is primarily an inflammatory disease characterized by the accumulation of macrophage foam cells in the vascular wall, triggering the secretion of numerous inflammatory mediators and leading to the recruitment of other immune cells (50). One mechanism by which *trans* fatty acids may promote atherogenesis is by activating inflammation (36). In a randomized controlled trial in healthy men, a diet containing 8% of daily energy from industrial *trans* fatty acids caused a 3.4-fold increase in plasma concentrations of C-reactive protein (CRP) after 5 wk of intake compared to a control diet with no *trans* fatty acids (51). In subjects with moderate hypercholesterolemia, the consumption of industrial *trans* fatty acids from stick margarine at 6.7% of total energy for 32 d significantly increased concentrations of TNFα, IL-1β and IL-6 in peripheral blood mononuclear cells (PBMCs) compared with PUFAs from soybean oil (52). Furthermore, in 2 independent cross-sectional studies, 1 conducted in overweight women (53) and the other in patients with heart disease (54), the intake of foods rich in industrial *trans* fatty acids positively correlated with plasma concentrations of inflammatory markers such as CRP, TNFα, chemokine (C-C motif) ligand 2 (CCL2), and IL-6, after adjustment for various factors.

**FIGURE 2:**
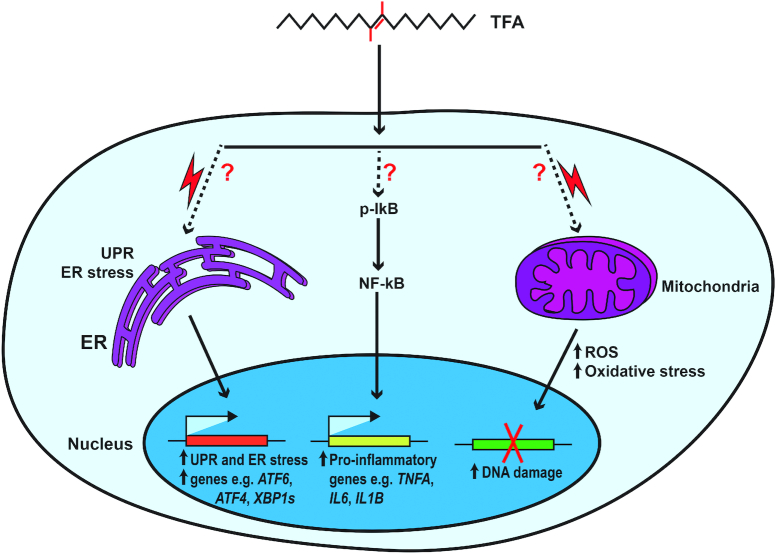
Proposed molecular mechanisms of *trans* fatty acids. *ATF4*, activating transcription factor 4; *ATF6*, activating transcription factor 6; ER, endoplasmic reticulum; p-IκB, phosphorylated IκB; ROS, reactive oxygen species; TFA, *trans* fatty acid; UPR, unfolded protein response; *XBP1s*, X-box binding protein 1, spliced form; ?, represents unclarified mechanism.

Due to ethical constraints and other challenges connected with human studies, mechanistic probing into the proinflammatory effects of *trans* fatty acids has been conducted in animal models and via in vitro experiments. Using atherosclerosis-prone LDL-receptor knockout mice, it was shown that compared with mice fed a diet rich in PUFAs, 16 wk of diets enriched with elaidic acid from partially hydrogenated soybean oil significantly increased the release of the inflammatory cytokine IL-6 by ∼1.5-fold (48), as well as the expression of *Ccl2, Tnfa*, and *Il-6* by ≥2-fold in abdominal aortas (49). Secondary to the induction of inflammation, the mice on the *trans* fatty acid diet showed increased accumulation of activated macrophages in the enlarged atherosclerotic lesions in the aortic intima (48, 49). Another animal study reported that ad libitum feeding of C57BL/6 mice for 16 wk with a *trans* fatty acid diet, providing ∼13% of energy intake from partially hydrogenated vegetable oil, resulted in a hepatic necroinflammatory phenotype, characterized by a 4.4-fold increase in hepatic *Tnfa* expression compared with a control diet (55). The proinflammatory effect of *trans* fatty acids seems robust and transcends specific animal models. In peroxisome proliferator–activated receptor (PPAR) α–deficient mice (56), as well as in mice with full body or liver-specific knockout of 11β-hydroxysteroid dehydrogenase type 1 (11β-HSD1) (57), *trans* fatty acid-enriched diets enhanced activation of NF-κB and increased hepatic expression of *Tnfa, Ccl2, Opn* (osteopontin), and macrophage markers (56, 57). A number of in vitro studies have examined the mechanism of action of *trans* fatty acids using different cell types. Please note that the concentration of fatty acids used in all in vitro studies included in this review ranges from 0.05 to 0.5 mM, with some exceptions, which can be considered physiological, as the total plasma *trans* fatty acid concentrations in humans can reach as high as 0.6 mM and 0.09 mM in nonfasting (58) and fasting (59) conditions, respectively. At the cellular level, the activation of the transcription factor NF-κB by the industrial *trans* elaidic and linoelaidic acids but not *cis*-linoleic acid was demonstrated in human microvesicular endothelial cells. Compared with untreated controls and linoleic acid, 0.05 mM and 0.1 mM concentrations of elaidic or linoelaidic acid resulted in profound activation of NF-κB signaling, demonstrated by an ∼2-fold increase in IκB-α phosphorylation and subsequent elevation in IL-6 concentrations and *TNFα* expression (60). So far, the induction in NF-κB signaling is the most plausible mechanism by which *trans* fatty acids stimulate inflammation, even though the experimental studies supporting this hypothesis are very few and do not interrogate this pathway in great detail (**Figure 2**). In a recent study, Hirata and colleagues showed that in comparison to both control and oleic acid, 12-h incubation with 0.2 mM elaidic acid induced cleavage of caspase 3 leading to enhanced apoptotic cell death in RAW264.7 macrophages. The elaidic acid effect was mediated through hyperactivation of the apoptosis signal-regulating kinase 1 (ASK1)-p38 mitogen-activated protein (MAP) kinase pathway, which is reported to promote inflammatory signal transduction (61).

Whereas the above studies point to proinflammatory effects of *trans* fatty acids, a number of other studies have reported neutral or anti-inflammatory effects of certain *trans* fatty acids. We recently showed that in angiopoietin-like 4 (ANGPTL4)-deficient C57BL/6 mice, a 7-wk intervention with a high-fat diet supplying ∼14% of calories as *trans* fatty acids from partially hydrogenated soybean oil did not induce inflammation compared with an isocaloric diet containing SFAs. Mice deficient in ANGPTL4, an endogenous inhibitor of lipoprotein lipase, are unique in that they develop a massive acute phase response upon consumption of a diet rich in SFAs. The SFA-enriched diet markedly increased hepatic expression of serum amyloid A (SAA), haptoglobin, and lipocalin along with a concomitant elevation in the plasma concentrations of these inflammatory markers by ≥50-fold, whereas the *trans* fatty acid-enriched diet did not (62). Corroborative data were obtained in vitro in RAW264.7 macrophages incubated for 6 h with 0.5 mM fatty acids. Compared with the vehicle control, elaidic acid had a neutral effect or modestly decreased expression of inflammatory markers, whereas palmitic acid and stearic acid induced the expression of inflammatory markers by 2 or more fold, leading to increased protein concentrations of inflammatory and apoptotic markers (62). Accordingly, whether *trans* fatty acids should be classified as proinflammatory may depend on the reference, e.g., no treatment, *cis*-unsaturated fatty acids, or SFAs. Notwithstanding these considerations, experimental data from clinical studies, along with animal and cell culture experiments, suggest that the deleterious effects of *trans* fatty acids may be partly mediated by activating inflammation, especially when compared with *cis*-unsaturated fatty acids.

## ER Stress, Reactive Oxygen Species Production, and Oxidative Stress

At the cellular level, environmental, physiological, and pathological insults can generate varying degrees of stress, such as ER and oxidative stress. ER stress impairs ER function and leads to activation of the unfolded protein response (UPR). The UPR comprises a signaling cascade that promotes cell survival but can also lead to apoptotic cell death (63). High concentrations of reactive oxygen species (ROS) due to an imbalance between ROS production and antioxidant defense can result in oxidative stress and subsequent damage to lipids, DNA, and protein (64, 65).

When present in excessive amounts, certain fatty acids, including *trans* fatty acids, are known to be injurious to cells due to their modulatory effects on ER stress, ROS production, and oxidative stress (35). In a mouse model of hyperlipidemia, 8 wk of feeding a diet containing 5% elaidic acid significantly increased superoxide production compared with a diet containing 5% oleic acid, concomitant with increased atherosclerotic lesion size and increased NADPH oxidase expression in the aortic vessel wall (66). The authors confirmed the in vivo findings in cultured smooth muscle cells incubated for 24 h with 0.1 mM elaidic acid to further underscore the role of ROS and oxidative stress as mediators of *trans* fatty acid-induced atherosclerosis (66). In C57Bl6/J mice (67) and Wistar rats (68), the presence of industrial *trans* fatty acids in the diet resulted in hepatic lipotoxicity characterized by an increase in oxidative stress and a decrease in hepatic antioxidant activity of catalase, superoxide dismutase, and glutathione peroxidase.

Events in the endothelial lining of blood vessels are known to be important in the pathogenesis of vascular diseases. Human umbilical vein endothelial cells incubated for 24 h with elaidic and linoelaidic acid induced apoptosis through increased ROS production and raised the activity of caspase 3 in a dose-dependent manner from 0.1 to 1.0 mM (69). Furthermore, in human microvascular endothelial cells, a 3-h treatment with elaidic and linoelaidic acid at 0.05 mM and 0.1 mM increased superoxide production leading to vascular inflammation through enhanced NF-κB signaling (60). One study explored the possible mechanisms behind the neurotoxic effects of elaidic acid in SH-SY5Y neuroblastoma cells, which were incubated for 24 h with vehicle control or increasing concentrations of elaidic acid ranging from 0.01 mM to 0.8 mM. Along with increased ER stress, elaidic acid concentrations higher than 0.1 mM induced ROS production and decreased the activity of antioxidants, which resulted in oxidative damage and apoptosis (70).

By contrast, in RAW264.7 macrophages treated with 0.5 mM fatty acids for 24 h, elaidic acid showed a similar inability to oleic acid to induce ER stress. Whereas palmitic acid potently induced ER stress characterized by enhanced mRNA and protein expression of several stress markers, including C/EBP homologous protein (CHOP) and spliced activated form of X-box binding protein 1 (XBP1s), elaidic acid had only a modest to no effect (62). Taken together, there is some evidence suggesting that *trans* fatty acids promote oxidative and ER stress when compared with *cis*-unsaturated fatty acids, which may contribute to their deleterious effects.

## Autophagy

Autophagy describes an adaptive response to stress during which organelles and other intracellular components are degraded in the lysosome as a way of recouping energy substrates for cell survival and maintenance of cell integrity. Autophagy involves a complex signaling cascade ranging from phagophore formation to the proteolytic degradation of engulfed targets by lysosomal proteases (71, 72). Few studies have investigated fatty acids as triggers of autophagy (73). Incubation of primary cardiac myofibroblasts with 0.2 mM and 0.4 mM *trans*-vaccenic and elaidic acid for 24 h induced autophagy, as shown by autophagosome formation, microtubule-associated protein 1A/1B-light chain (LC) 3B (LC3-β) lipidation, LC3-II formation, and autophagy-related proteins 5-12 (ATG5-12) accumulation, leading to apoptosis, as reflected by elevated concentrations of cleaved caspases 9, 3, 7, and enhanced translocation of Bcl-2-associated X protein (Bax) to the mitochondria (74). These results indicate that *trans* fatty acids are potent stressors to myofibroblasts culminating in premature apoptosis. A recent study, however, reported contrasting results in U2OS cells stably expressing different biosensor markers of autophagy, UPR, and Golgi stress. In these cells, 6-h incubation with 0.5 mM elaidic or *trans*-vaccenic acid inhibited autophagy induced by SFAs (75). The suggestion was raised that the lipotoxicity of *trans* fatty acids may reside in their ability to inhibit the cytoprotective stress response induced by SFAs (75). So far, it is rather difficult to make tangible conclusions from the few available studies. Additional work is needed to clarify the exact role of *trans* fatty acids in autophagy and the implications for cardiometabolic health.

## Molecular Effects of *trans* Fatty Acids on the Liver

In the liver, an intricate balance exists between the uptake, storage, synthesis, secretion and oxidation of lipids in order to maintain local and systemic lipid homeostasis. Aberrant hepatic lipid metabolism can lead to dyslipidemia and nonalcoholic fatty liver disease (NAFLD), both of which are important risk factors for cardiovascular disorders (76, 77). A number of studies have shown that the liver bears the brunt of *trans* fatty acid-mediated pathology. In mice, the intake of diets providing 12–20% of energy as industrial *trans* fatty acid over a period of 7 to 24 wk was shown to promote liver damage, characterized by elevated concentrations of plasma alanine aminotransferase activity and increased plasma concentrations of acute phase proteins, including SAA and haptoglobin (55, 78–80). Furthermore, histological staining of liver slices showed profound fat accumulation in mice fed diets rich in industrial *trans* fatty acids as well as histological indications of nonalcoholic steatohepatitis and cirrhosis (55, 56, 81, 82). In a number of animal studies, the steatotic liver phenotype was associated with increased expression of genes involved in lipogenesis, including fatty acid synthase (*Fasn*), acetyl-CoA carboxylase α (*Acaca*), and sterol regulatory element binding protein (*Srebp1*) (56, 80, 82). In a recent study, we found that a 7-wk intervention with a diet supplying ∼14% of calories as *trans* fatty acids from partially hydrogenated soybean oil increased the ratio of liver to gonadal fat mass by ∼2-fold, plasma alanine aminotransferase activity by ∼3-fold, acute phase proteins haptoglobin by ∼10-fold, and SAA by ∼2-fold in comparison with diets enriched in *cis*-unsaturated fatty acids and SFAs. Additionally, the diet rich in industrial *trans* fatty acids increased steatosis, hepatic cholesterol concentrations, and fibrosis markers, suggesting enhanced NAFLD (78).

In conjunction with these studies in mice, several studies have examined the effect of elaidic acid on lipid metabolism in human and murine hepatoma cells. In human Huh-7 cells, compared with oleic acid and nontreated controls, treatment with 0.1 mM elaidic acid for 16 h stimulated SREBP1c-dependent lipid synthesis through enhanced expression of SREBP target genes, leading to an ∼2-fold increase in de novo synthesis of cholesterol and fatty acids (83). Luciferase reporter gene assays indicated that elaidic acid potently induced sterol regulatory element (SRE)-luciferase activity in HEK293 cells, whereas oleic acid inhibited SRE activity (83).

Nielsen and colleagues applied an integrated lipidomics, transcriptomics, and proteomics approach in human HepG2 cells incubated with 0.1 mM fatty acids for 24 h. Elaidic acid, but not oleic or stearic acid, potently increased the expression of key enzymes involved in cholesterol and fatty acid biosynthesis. Relative to oleic acid, elaidic acid upregulated SREBP2 expression by 2.3-fold along with increased expression of several other cholesterogenic genes such as HMG-CoA reductase by 2.9-fold, squalene epoxidase by 3.3-fold, and mevalonate kinase by 2.2-fold (84). We confirmed the findings of Nielsen and colleagues in murine Hepa1–6 hepatocytes treated for 24 h with 0.5 mM fatty acids, and showed an unequivocal role of SREBP2 in mediating the effect of *trans* fatty acids on lipid/cholesterol metabolism (78). Specifically, we found that elaidic acid potently upregulates the expression of genes involved in cholesterol synthesis and induces the expression and activity of SREBP2. Silencing of *Srebp2* by siRNA-mediated knockdown abrogated the cholesterogenic effect of elaidic acid. The *Srebp2* activation by elaidic acid is likely due to lowered concentrations of intracellular free cholesterol since cholesterol is a negative regulator of the SREBP signaling pathway. A previous study reported that elaidic acid is a high affinity substrate for the esterification of cholesterol into cholesterol esters (85), which might explain why elaidic acid reduces concentrations of intracellular free cholesterol. In support of this hypothesis, elaidic acid was shown to increase the expression of the cholesterol esterification enzyme, sterol *O*-acyltransferase 1 (*Soat1*), by 2.1-fold compared with oleic acid (84). In our study, although elaidic acid significantly lowered intracellular concentrations of free cholesterol by almost half, there was no concomitant increase in concentrations of cholesterol ester compared with the nontreated control (78). Furthermore, silencing of *Soat1* did not abrogate the induction of cholesterol synthesis genes by elaidic acid. Accordingly, enhanced cholesterol esterification likely does not explain the increase in SREBP2 activity by elaidic acid. Rather, elaidic acid appears to decrease the sensitivity of SREBP cleavage-activating protein (SCAP) to cholesterol (78). Overall, the cholesterogenic effect of elaidic acid can at least partly be ascribed to its ability to reduce the concentrations of intracellular free cholesterol and decrease the sensitivity of SCAP to cholesterol. This anabolic effect of elaidic acid may contribute to the deleterious effect of industrial *trans* fatty acids on lipid metabolism.

## Molecular Effect of *trans* Fatty Acids on Adipose Tissue

Compared with the liver, fewer studies have reported the effects of *trans* fatty acids on adipose tissue. Most human studies that have investigated the effects of *trans* fatty acids on adipose tissue have focused on conjugated linoleic acids (CLAs). CLAs are a distinct class of naturally occurring *trans* fatty acids that require their own full review, as done elsewhere (86, 87), and therefore will only be discussed briefly here. In the USA, 10E,12Z CLA is marketed commercially as a natural weight loss supplement due to its reported ability to reduce adipose tissue mass (88, 89). Notwithstanding their potential health benefits, a number of studies have revealed that intake of CLAs may cause negative side effects such as increasing plasma markers of inflammation and oxidative stress (88), liver damage (90), and inflammation in macrophages (91). In mice, diets that contain 10E,12Z CLA remarkably reduced fat mass yet at the same time promoted hyperinsulinemia, adipose tissue inflammation, and liver damage (92–95). Treatment of 3T3-L1 adipocytes with 0.25 mM 10E,12Z CLA for 7 d stimulated lipolysis and fatty acid oxidation, leading to enhanced lipid utilization and decreased triglyceride storage, when compared with 9Z,11E CLA, palmitic acid, or the nontreated control (96). At the molecular level, 10E,12Z CLA reduced the expression of lipogenic genes such as *Acaca*, diacylglycerol O-acyltransferase 1 and 2 (*Dgat1* and *Dgat2*), by ≥2-fold, and increased the expression of the fat oxidation gene, carnitine palmitoyltransferase 1A (*Cpt1a*), by ∼3.5-fold compared with the nontreated control. Interestingly, the increased fatty acid oxidation in adipocytes was associated with increased mitochondrial ROS production and a proinflammatory response, characterized by the increased expression of *Ccl2* and *Il6* (96).

Apart from CLAs, a diet enriched with elaidic acid reduced fat tissue mass in LDL-receptor knockout mice along with an increase in liver mass and liver steatosis (82). Consistent with these findings, and as previously mentioned, feeding wild-type C57BL/6 mice a high-fat diet rich in industrial *trans* fatty acids reduced adipose tissue mass by ∼30% but increased liver mass by ∼40% compared with isocaloric diets rich in *cis*-unsaturated fatty acids or SFAs (78). The decrease in adipose tissue mass was accompanied by a strong upregulation of genes involved in fatty acid and cholesterol synthesis in both gonadal and inguinal fat tissue depots (78, 97). The evidence from these studies suggests that diets rich in *trans* fatty acids cause preferential fat accumulation in the liver at the expense of adipose tissues. The adipose tissue seems to compensate for this anomaly by stimulating de novo lipogenesis. The molecular mediators accounting for the preferential fat trafficking to the liver remain unknown. These intriguing observations warrant further investigation, as any mechanistic insight gained could be utilized to redirect fat towards specific tissues under different physiological states to reduce ectopic fat accumulation and potentially diminish NAFLD.

## Mechanisms of Action of Industrial and Ruminant *trans* Fatty Acids

There is an ongoing debate as to whether industrially produced and naturally produced ruminant *trans* fatty acids exert the same effects on cardiovascular health. A number of human studies have reported that the harmful effects of *trans* fatty acids are exclusive to industrial *trans* fatty acids (1, 31, 98), whereas ruminant *trans* fatty acids are reported to be innocuous or even beneficial to cardiovascular health (99–101). In contrast, other epidemiological and clinical studies have reported that ruminant *trans* fatty acids are equally culpable as industrial *trans* fatty acids in promoting cardiovascular diseases (102–105). Below we will delineate the possible differences between industrial and ruminant *trans* fatty acids at the molecular level.

With respect to plasma lipoproteins, Gebauer and colleagues compared the effects of isocaloric diets containing 3.3% energy as either stearic acid (control diet), *trans*-vaccenic acid, or industrial *trans* fatty acids in a randomized, crossover feeding trial in 106 healthy subjects who were each provided the diets for 24 d. Compared with the control diet, the industrial *trans* fatty acid diet increased total and LDL cholesterol by 1.9% and 3.4%, respectively, whereas the ruminant *trans* fatty acid diet resulted in the greatest increase in total and LDL cholesterol by 4.5% and 6.1%, respectively, leading to the conclusion that both vaccenic acid and *trans* fatty acids derived from partially hydrogenated oil adversely affect LDL cholesterol. Furthermore, the ruminant *trans* fatty acid diet marginally increased HDL cholesterol by 2.1%, whereas the industrial *trans* fatty acid diet had no significant effect on HDL cholesterol (105). Brouwer and colleagues conducted a quantitative review in which they examined the effects of industrial and ruminant *trans* fatty acids in humans. To eliminate differences in control treatments and allow for comparison between the studies, for each study the authors recalculated what the effect of *trans* fatty acid on lipoprotein would be if they isocalorically replaced *cis* MUFAs. For each percentage of dietary energy, linear regression analysis showed that industrial and ruminant *trans* fatty acids both increased the plasma ratio of LDL to HDL cholesterol by 0.055 and 0.038, respectively (104). The above studies demonstrate that when the consumption levels of ruminant and industrial *trans* fatty acids are matched, they have similar effects on plasma lipoproteins.

Differences in biological mechanisms between ruminant and *trans* fatty acids have been examined using preclinical models in animals and cultured cells. In 1 study, LDL-receptor knockout mice were fed for 14 wk with a diet containing either 4% partially hydrogenated vegetable shortening providing 1.5% *trans* fatty acids in the form of elaidic acid, or 15% butter providing 1.5% ruminant *trans* fatty acids in the form of vaccenic acid. Compared with a control diet that contained no *trans* fatty acids, the elaidic acid diet increased atherosclerotic plaque formation by ∼5% of the aortic luminal surface area, whereas the vaccenic acid diet increased the plaque size by <1%. When each of the diets was supplemented with 2% cholesterol, the relative atheroprotective effect of vaccenic acid was potentiated as the vaccenic acid diet significantly reduced atherosclerotic plaque formation by 6.2% of the aortic luminal surface area, whereas the elaidic acid diet showed no significant changes in comparison to the control diet (106). Hence, although ruminant and industrial *trans* fatty acids appear to have similar effects on plasma lipoprotein concentrations in humans, the few studies in animal models suggest a protective effect of ruminant *trans* fatty acids against cardiovascular disease. This highlights the importance of other potential mechanistic differences with respect to inflammation, ROS production, ER stress, and oxidative stress, as previously discussed.

In cultured cells, ruminant *trans*-vaccenic and palmitoleic acid but not elaidic acid potently reduced mRNA expression of *TNFA* in a dose-dependent manner in both HUVEC and HepG2 cells compared with the vehicle control (107). A similar study by Iwata and colleagues showed that a 3-h treatment of ≤0.1 mM elaidic acid activated NF-κB signaling and increased superoxide production in microvesicular endothelial cells, whereas *trans*-vaccenic acid showed no such response (60). It was suggested that the anti-inflammatory effect of ruminant *trans* fatty acids may be due to their property as better ligands for PPARγ. *trans*-Vaccenic acid was shown to activate the transcriptional activity of PPARγ and/or PPARα in the JCR:LA-cp rat model of dyslipidemia and metabolic syndrome (108–110) and in PBMCs (111). In human PBMCs, incubation with 0.1 mM vaccenic acid for 19 h significantly decreased the intracellular content of IL-2 and TNFα in T-helper cells by 38% and 31%, respectively, compared with the untreated control. The vaccenic acid effect was dependent on PPARγ since the presence of the PPARγ antagonist, T0070907, restored the IL-2 and TNFα-positive T-helper cell population (111). Although the number of studies is limited, the molecular effects of ruminant *trans* fatty acids may be mediated by PPARs, whereas industrial *trans* fatty acids regulate transcription via SREBPs, leading to an increase in lipo- and cholesterogenic gene expression. Indeed, in HepG2 cells, elaidic acid increased protein concentrations of enzymes involved in cholesterol synthesis and transport, whereas *trans*-vaccenic acid did not (112). Similarly, in Hepa1–6 and 3T3-L1 adipocytes, 24-h incubation with 0.5 mM elaidic acid distinctly stimulated activity of SREBP2 leading to induced expression of cholesterol and fatty acid synthesis genes, which was not observed with ruminant *trans* fatty acids including vaccenic acid (78).

Other pathways, such as cytotoxicity and inflammation, may be similarly regulated by industrial and ruminant *trans* fatty acids. In RAW264.7 macrophages, both elaidic acid and *trans*-vaccenic acid induced apoptotic cell death and inflammation (61). In a separate study in RAW264.7 macrophages treated for 24 h with fatty acids, palmitic acid potently induced gene markers of inflammation and ER stress, which could not be reproduced with either industrial or ruminant *trans* fatty acids (62). With respect to autophagy, a study has juxtaposed the effects of industrial and ruminant *trans* fatty acids by incubating U2OS cells for 6 h with 0.5 mM elaidic or *trans*-vaccenic acids, showing that both *trans* fatty acids similarly inhibited autophagy induced by SFAs (75).

Taken together, evidence from both preclinical and clinical studies show that industrial and ruminant *trans* fatty acids can behave similarly or differentially, depending on the biological pathway and clinical parameters under investigation. As mentioned earlier, it has been suggested that the alleged positive effects of ruminant *trans* fatty acids could be due to a relatively low intake compared with industrial *trans* fatty acids. Nonetheless, the underlying mechanisms for the reported differences are still unclear. From a biochemical perspective, the differences in the source and slight change in the position of the *trans* double bond may not justify the separate classification of industrial and ruminant fatty acids. However, there are numerous examples of bioactive molecules in which a slight change in molecular structure has a profound impact on the biological properties. Indeed, the position of the *trans* double bond could impact the extent to which fatty acids are taken up, sensed, metabolized, and incorporated into cellular organelles and membranes. Such potential mechanisms are still poorly delineated and warrant further investigation.

## Physiological Relevance of Animal and In Vitro Studies to Humans

The direct relevance of many animal and in vitro studies to humans is certainly debatable. This review has explored mechanistic insights of *trans* fatty acids from studies in mice and in vitro models. As in humans, mice do not synthesize *trans* fatty acids endogenously but obtain all *trans* fatty acids from the diet. Therefore, animal studies can be modeled to mimic the levels of intake in humans, thereby improving the extrapolation of the results to humans. The dosage of *trans* fatty acids used in the animal experiments described in this review varied from 1.5% to 20% of total energy. At the lower end, this dosage is representative of the physiological levels of intake in humans in countries that do not legally restrict the amounts of industrial *trans* fatty acids in food. However, even in countries that have restrictive laws, excessive consumption of certain foods could lead to intake levels of *trans* fatty acids approaching levels reported in the animal experiments. With respect to the in vitro studies, the concentration of fatty acids used ranges from 0.05 mM to 1 mM. The total *trans* fatty acid concentration in blood plasma can reach as high as 0.6 mM but is expected to be much lower in most individuals and depends on the intake level. The use of *trans* fatty acid concentrations above 0.5 mM is likely supraphysiological.

Lipoprotein metabolism is well-known to be different between mice and humans. Whereas cholesterol circulates mainly in the form of the proatherogenic LDL particles in humans, in mice cholesterol mainly circulates in the form of HDL (113). In addition, unlike humans, wild-type mice do not develop spontaneous atherosclerosis. Therefore, in this review, discussions on the effect of *trans* fatty acids on plasma lipoproteins have focused on human studies or in specific mouse models such as the LDL-receptor knockout mice, which carry similar lipoprotein profiles as humans and develop atherosclerosis (114, 115).

## Conclusions

Epidemiological studies have indicated that a higher intake of industrial *trans* fatty acids is associated with an increased risk of cardiovascular disease. Clinical studies in humans have shown that this association is likely explained by an increase in total and LDL cholesterol concentrations and a decrease in HDL cholesterol concentrations by industrial *trans* fatty acids. Preclinical studies in mice and cultured cells have partly clarified the molecular mechanisms of action of industrial *trans* fatty acids. In cultured cells, industrial *trans* fatty acids stimulate inflammation, ER stress, and oxidative stress, although less potently than SFAs. Besides impacting on inflammation and stress-related pathways, industrial *trans* fatty acids have a profound influence on lipid metabolism. In mice, industrial *trans* fatty acids promote the preferential storage of fat in the liver at the expense of fat tissue. Studies in cultured liver cells indicate that industrial *trans* fatty acids stimulate the cholesterol synthesis pathway by activating SREBP2-mediated gene regulation.

Overall, the reported distinct effects of industrial and ruminant *trans* fatty acids in vitro and in vivo prevents the grouping of all *trans* fatty acids as a single entity. From an evolutionary perspective, certain animals have conserved the ability to endogenously synthesize certain types of *trans* fatty acids, rendering it unsurprising that such ruminant *trans* fatty acids may carry certain benefits. Presently, there is still limited understanding of the mechanism of action of *trans* fatty acids. Accordingly, further mechanistic studies are required to fully clarify both defined and undefined properties of industrial and ruminant *trans* fatty acids. Gaining a deeper understanding of the molecular mechanism of action of *trans* fatty acids may create new therapeutic windows for the treatment of diseases characterized by disrupted lipid metabolism.

## Notes

Supported by funding from the Graduate School, Voeding, Levensmiddelentechnologie, Agro-Biotechnologie en Gezondheid (VLAG), from Wageningen University, The Netherlands.

Author disclosures: The authors report no conflicts of interest.

Abbreviations used: ACACA, acetyl-CoA carboxylase α; ANGPTL4, angiopoietin-like 4; CCL2, chemokine (C-C motif) ligand 2; CLA, conjugated linoleic acid; CRP, C-reactive protein; ER, endoplasmic reticulum; LC, light chain; NAFLD, nonalcoholic fatty liver disease; PBMC, peripheral blood mononuclear cell; PPAR, peroxisome proliferator–activated receptor; ROS, reactive oxygen species; SAA, serum amyloid A; SCAP, SREBP cleavage-activating protein; SFA, saturated fatty acid; SOAT1, sterol *O*-acyltransferase 1; SRE, sterol regulatory element; SREBP, sterol regulatory element binding protein; UPR, unfolded protein response.
